# Perceived quality of physiotherapist-led orthopaedic triage compared with standard practice in primary care: a randomised controlled trial

**DOI:** 10.1186/s12891-016-1112-x

**Published:** 2016-06-10

**Authors:** Karin S. Samsson, Susanne Bernhardsson, Maria E. H. Larsson

**Affiliations:** Department of Health and Rehabilitation, Institute of Neuroscience and Physiology at Sahlgrenska Academy, University of Gothenburg, Box 430, 405 30 Gothenburg, Sweden; Närhälsan Tjörn Rehabilitation Clinic, Primary Health Care, Region Västra Götaland, Syster Ebbas väg 1, 471 94 Kållekärr, Sweden; Närhalsan Research and Development Primary Health Care, Region Västra Götaland, Kungsgatan 12, 6th floor, 411 18 Gothenburg, Sweden

**Keywords:** Advanced practice physiotherapy, Physical therapy, Quality of care, Patient perception, Sweden, Expectations

## Abstract

**Background:**

Physiotherapist-led orthopaedic triage, where physiotherapists diagnose and determine management plans, aims to enhance effectiveness and provide the best care. However, scientific evidence for the effectiveness of this model of care remains limited, and there are few studies reporting on patients’ perceptions of the care provided. The purpose of this study was to evaluate patients’ perceived quality of care in a physiotherapist-led orthopaedic triage in primary care, compared with standard practice.

**Methods:**

In a randomised controlled trial, patients of working age referred for orthopaedic consultation at a primary healthcare clinic in Sweden received either physiotherapist-led triage (*n* = 102) or standard practice (orthopaedic surgeon assessment) (*n* = 101). Neither subjects nor clinicians were blinded.

The questionnaire Quality from the Patient's Perspective (QPP) was used to evaluate perceived quality of care focusing on the caregivers’ medical-technical competence and identity-orientated approach. Also, to what extent patients’ expectations were met, and their intention to follow advice was evaluated.

**Results:**

For this study, 163 patients (80 %) were analysed (physiotherapist-led triage (*n* = 83), standard practice (*n* = 80)). Participants perceived significantly higher quality of care with the triage than with the standard practice in regards to receiving best possible examination and treatment (medical-technical competence) (*p* < 0.001). This was also found in regards to receiving information about examination and treatment (*p* < 0.001), results (*p* < 0.001), and self-care (*p* < 0.001), the caregiver’s understanding (*p* < 0.001), respect (*p* < 0.001) and commitment (*p* < 0.001) as well as the opportunity to participate in decision-making (*p* = 0.01) (identity-orientated approach). Participants in the physiotherapist-led triage group reported to a significantly higher extent that their expectations of the treatment were met (*p* < 0.001), as well as the intent to follow the advice and instructions received (*p* = 0.019).

**Conclusions:**

This paper reports on patients’ perceptions of quality of care in a physiotherapist-led orthopaedic triage compared with standard practice. Patients in both groups reported that they perceived good quality of care, with the patients in the physiotherapist-led triage reporting significantly higher perceived quality of care than those in the standard practice group. This model of care seems to meet patients’ expectations and result in a greater intention to follow advice and instructions for self-management.

Our findings are in line with existing literature that this model of care provides an opportunity to shape patient-centered care that can improve access and offer care on the most appropriate level, with maintained good quality of care.

**Trial registration:**

Clinical Trials NCT02265172. Registered 10 June 2014

## Background

Considering the increasing demand on health care to provide accurate and timely diagnosis and care for patients with musculoskeletal complaints, alternative models of care, such as physiotherapist-led orthopaedic triage, have been explored predominantly in the UK, Australia and Canada [1–10]. The aims of triage have been suggested to be to reduce long waiting times and to enhance effectiveness and best care/practice, i.e. timely access to the right care from the appropriately qualified health care professionals who can direct patients towards the optimal treatment pathway [11]. Physiotherapists have been used to triage, diagnose, and determine management plans, and to refer for investigations, orthopaedic surgery or conservative management [3, 12, 13]. Studies of this model of care, in various outpatient settings, have reported high agreement on diagnosis and treatment approach between physiotherapists and orthopaedic surgeons [4, 8, 10, 14, 15]. Several studies have shown that physiotherapist-led orthopaedic triage also decreases referrals for orthopaedic consultation [1, 5, 15–17]. Also, emerging evidence suggests that such a model of care can improve access to care with equal or better outcomes compared to standard practice (i.e. orthopaedic surgeon consultation) regarding waiting times, treatment effectiveness, use of healthcare resources, economic costs, both patient and provider satisfaction, and patient outcomes such as pain and functional disability [18, 19].

We have previously reported primary outcomes from a randomised controlled trial investigating physiotherapist-led orthopaedic triage in a Swedish primary healthcare setting [20]. The study showed significantly better selection accuracy and shorter waiting times with physiotherapist-led triage than with standard practice. Furthermore, the study showed no differences between physiotherapy-led triage and standard practice regarding the secondary outcome long-term follow-up of patient-reported outcome measures [21]. In this paper, we report on the secondary outcome patients’ perceived quality of care with this model of care.

Several studies have reported that patients are either equally [3, 6] or even more satisfied with physiotherapist-led triage than with standard practice [4, 8]. Still, scientific evidence for the effectiveness of this model of care remains limited and there is a scarcity of high-quality studies [22]. Few of the studies report on health outcomes using standardised outcome measures [11]. Additionally, considering differences among national healthcare systems, studies of such a model need to be conducted in each respective country [23].

Good care should be patient-centred [24], which is characterised by respectful and individualised care. One of the core components of patient-centred care is participation in clinical decision-making, which empowers patients to actively engage in their care [25]. Therefore, patients’ perceptions of the quality of their health care are of increasing interest for healthcare policy makers [24, 26]. Additionally, evaluating the patients’ perceptions is essential to any new role involving a shift in traditional practice boundaries [27].

Wilde et. al. [28] have presented a model that stipulates that patients’ perceptions of what constitutes quality of care are formed by their encounters with an existing care structure, and by their norms, expectations, and experience. Based on this model, the questionnaire Quality from the Patient’s Perspective (QPP) was developed.

Considering that in many countries it is still standard practice for patients to be assessed by orthopaedic surgeons when referred for orthopaedic consultation, we were interested in exploring patients’ perceptions of the quality of care of a physiotherapist-led triage. Even though previously reported results indicate that physiotherapist-led orthopaedic triage could provide appropriate care for the patients, patients’ perceptions of this model need to be investigated before large scale implementation. Therefore, the purpose of this paper was to evaluate patients’ perceived quality of care in a physiotherapist-led orthopaedic triage in primary care compared with standard practice. Furthermore, we wanted to evaluate outcome-related aspects: whether patients’ expectations were met, and patients’ intention to follow advice and instructions.

## Methods

### Study design

This paper reports findings from a randomised controlled trial, and the full study design and method of this trial have been reported previously [20, 21]. The hypothesis of the trial was that this new model of care would provide good selection accuracy, without negatively affecting patient-related outcomes and while maintaining good quality of care. From this trial, two papers have been previously published: one reported on the primary outcome selection accuracy for orthopaedic intervention [20], and one reported long-term effects on patient-reported outcomes [21].

### Setting and participants

The study took place at a primary healthcare centre in a Swedish municipality. Consecutive recruitment of patients referred for orthopaedic surgeon consultation at the healthcare centre was performed between August 2009 and January 2011 with the following inclusion criteria: working age (between 18 and 67 years of age), sub-acute (four weeks to three months) or persistent (>three months) musculoskeletal pain, and the ability to understand written and spoken Swedish. The exclusion criteria were chosen in collaboration with the orthopaedic surgeon in the study. Patients were excluded if the stated diagnosis on the referral was hallux valgus, ganglion or trigger finger, where the general practitioners (GPs) were assumed to have high accuracy in diagnosis.

### Procedure

We used a block randomisation with a 1:1 allocation and block sizes of 20 to ensure an equal allocation ratio [29]. Sealed, opaque envelope containing details of the allocated group were mixed and put in a box by an administrator. After receiving verbal consent for participation, the administrator randomised the patient by drawing the next envelope from the box. Due to the nature of the intervention it was not possible to blind therapists or participants to their group allocation.

### Physiotherapist-led orthopaedic triage

The physiotherapist in this trial, also the first author of this paper, did not receive any training specific for this trial. She had specialist training in the form of postgraduate qualifications, including a master’s degree in Manipulative Therapy, one year of mentored clinical practice within the scope of orthopaedic manual therapy (OMT) and eight years of clinical experience in primary care, four within the scope of OMT. The duration of the appointment was up to 60 min with the main aims to diagnose and determine the most appropriate management pathway. The patients also received a brief treatment comprised of advice on ergonomics and/or exercises when needed; however, only during the one visit. Management pathways consisted of one or more of the following referrals; further investigation (i.e. x-rays, MRI), orthopaedic surgeon consultation (i.e. appropriate candidate for surgery), physiotherapy or occupational therapy (for conservative management with on-going support) or back to the patient’s GP. If patients were found to be appropriate candidates for surgery, the physiotherapist had the authority to make an appointment with the orthopaedic surgeon at the healthcare centre, without consideration of the waiting list. This was made to ensure that if the patients were considered appropriate for surgery, the total waiting time for consultation with the orthopaedic surgeon was not longer than the patients in the standard practice group due to their participation in the study. Referrals for further investigations were requested and sent via the patient’s GP and the images could be assessed together with the orthopaedic surgeon, if needed. One or two optional follow-up visits were offered when needed, for example follow-up after treatment or investigations.

### Standard practice

The orthopaedic surgeon in this trial had 26 years of experience in orthopaedic medicine, 21 of which were as an orthopaedic specialist. The duration of the appointment was 15 min, with the main aims to diagnose and determine the most appropriate management pathway. The patients received advice, prescriptions or injections, when needed. Management pathways were the same as for the triage group with the addition of orthopaedic intervention (i.e. minor surgery at the present healthcare centre), and referral to orthopaedic clinic for orthopaedic intervention (i.e. appropriate candidates for surgery). One or two optional follow-up visits were offered when needed, for example follow-up after investigations.

### Outcome measures

Patients’ perceptions of quality of care were assessed using the QPP. As per the choice of the participant, the questionnaire was sent either by post or as an online survey approximately five days after the appointment. Up to two reminders were sent. To reduce risk of bias, distribution and administration of the questionnaires were handled by an independent administrative company, ImproveIT (Halmstad, Sweden). The QPP is a self-administered questionnaire developed using a grounded theory approach and consisting of items formulated in words used by patients [28]. The questionnaire has been psychometrically tested [30, 31] and validated in different settings [32–34]. A short version of the questionnaire has been psychometrically tested [35–38]. The short, swedish version was used in this study, with minor modifications to include the physiotherapist and the orthopaedic surgeon. The QPP questionnaire is based on the assumption that patients’ perception of quality of care may be considered in four dimensions: caregivers’ medical-technical competence; care organisations’ physical-technical conditions; degree of identity-orientation in the caregivers’ attitudes and actions; and the care organisations’ socio-cultural atmosphere [32]. The primary outcomes reported in this paper were the following two dimensions (comprising eight items): *medical-technical competence* (one item) and *identity-orientated approach* (seven items regarding information, participation, and the caregiver’s commitment, understanding and respect). Each item is evaluated in two ways using a 4-point Likert scale; first the patients rate how they perceive the quality of care (PR; perceived reality);”This is what I experienced…”, ranging from 1 (Do not agree at all) to 4 (Completely agree). The patients then also rate how important that aspect of care is (SI; subjective importance);”This is how important it was to me…”, ranging from 1 (Little or no importance) to 4 (Of the very highest importance). Each item also has a “Not applicable” response option. The secondary outcomes were two additional items from the QPP measuring outcome-related aspects: “Will you follow the advice and instructions that you have now received from the physiotherapist/orthopaedic surgeon?” (response options rated on a 3-point Likert scale ranging from 1 (No) to 3 (Yes, completely), or Not applicable (Don’t know or I have not received any advice or instructions)), and “To what extent were your expectations of the treatment met?” rated on a 5-point Likert scale ranging from 1 (Not at all) to 5 (To a very large extent).

Demographic data including age, sex, civil status, country of birth, and education were collected to describe the study population.

### Sample size

Sample size was originally calculated based on the trial’s main outcome variable, selection accuracy, as previously reported [20]. To verify that the study had sufficient power to detect a difference in the secondary outcome, QPP, a retrospective power calculation was made. This was based on QPP mean scores for the item”I received the best possible examination and treatment (as far as I can tell)” (range 1-4). A relevant mean difference between groups for items from the QPP has been suggested to be 0.35 [39]. Analysis were made using an online calculator from University of British Columbia, Canada [40].

### Data analysis

Descriptive statistics for demographic characteristics were used and analysed with the Independent *t*-test or the Pearson’s Chi-squared test to determine any baseline differences between the groups. Between-group comparisons were made for the QPP data using the Mann-Whitney *U*-test, and medians, quartile 1 and 3, and means are reported. The significance level was set to *p* < 0.05.

Demographic data of responders vs. non-responders were analysed within each respective group using the Independent *t*-test and the Pearson’s Chi-squared test.

The QPP data were registered by Improve IT staff with the KUPPIT^1^ software (ImproveIT, Halmstad, Sweden, 2003). All collected data were transferred and analysed by the first author using IBM SPSS, version 18.0 (IBM Corp, Armonk, NY, USA).

## Results

### Participants

**Fig. 1:**
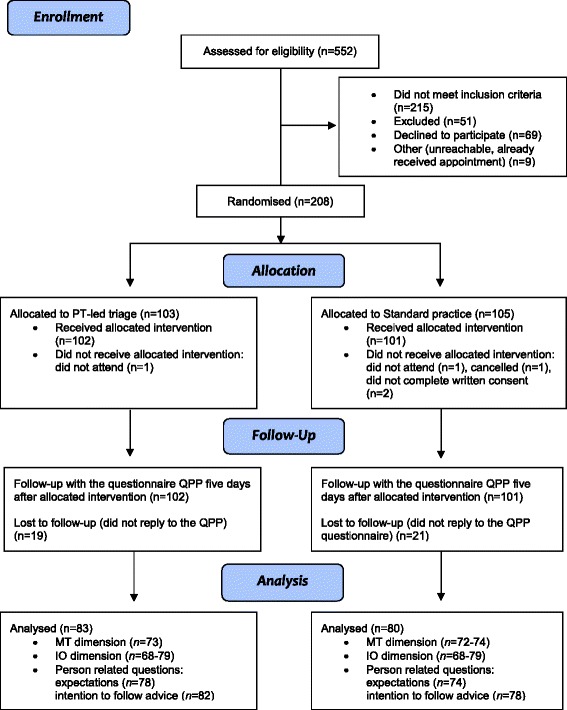
CONSORT flow chart of the progress of participants through the study. PT = physiotherapist, QPP = Quality from the patients’ perspective, MT = Medical-technical competence, IO = Identity-orientated approach

The inclusion and analysis process is illustrated in Fig. 1. The total response rate for the QPP was 80 % (81 % in the physiotherapist-led triage group and 79 % in the standard practice group). The reasons for non-response are unknown. There were no significant baseline differences between the two groups with respect to sex, civil status, and country of birth, education or occupation; however, participants in the standard practice group were significantly older (Table 1).

**Table 1:** Demographic characteristics of the respondents of the QPP questionnaire at baseline

	Physiotherapist-led triage *n* = 83 n (%)	Standard practice *n* = 80 n (%)
Age (years)^*^		
Mean	51	55
Range	18–67	21–67
SD	10.6	12.5
Sex		
Male	38 (46)	36 (45)
Female	45 (54)	44 (55)
Civil status		
Married/living together	69 (83)	73 (91)
Single/living alone	14 (17)	7 (9)
Country of birth		
Sweden	81 (98)	76 (95)
Other	2 (2)	4 (5)
Education		
Elementary school	10 (12)	19 (24)
Upper secondary school	38 (46)	33 (41)
University	35 (42)	28 (35)
Occupation		
Working	61 (73)	53 (67)
Student	2 (2)	2 (2)
Other	20 (24)	25 (31)

*QPP* Quality from the Patients’ Perspective, *SD* Standard Deviation

^*^Statistically significant difference between groups (*p* = 0.036)

### Study power

In this study, the mean difference for the item”I received the best possible examination and treatment (as far as I can tell)” (measured on a 1-4 scale), was 0.60 units (triage group mean 3.51 vs. standard practice group mean 2.91, SD 0.94), and with a sample size of 73 participants in each group (respondents to this particular item) we reached sufficient power (0.97) to detect differences at the *p* < 0.05 level.

### Quality of care from the patient perspective

As presented in Table 2 participants perceived significantly higher quality of care with the triage assessment than with the orthopaedic surgeon assessment; both regarding receiving best possible examination and treatment (medical-technical competence) and regarding receiving information, the opportunity to participate in decision making, and the caregiver’s understanding, respect and commitment (identity-orientated approach). Perceived importance of the different items was rated higher by patients in the triage group for the item in the Medical-technical competence dimension and for two of the items in the Identity-orientated approach dimension.

**Table 2:** Participants’ perceptions of the quality of care in the dimensions of medical-technical competence and identity-oriented approach

Dimension/factor		Physiotherapist-led triage *n* = 83						Standard practice *n* = 80				
		*n*	NA	Median	Q1;Q3	Mean	*n*	NA	Median	Q1;Q3	Mean	*p*-value
Medical-technical competence												
*Care received*												
I received the best possible examination and treatment.	PR	73	2	4	3; 4	3.5	74	4	3	2; 4	2.9	**<0.001**
	SI	73	2	4	3; 4	3.5	72	5	3	3; 4	3.3	**0.022**
Identity-oriented approach												
*Receiving information about*												
How examinations and treatments would take place.	PR	79	1	4	3; 4	3.6	69	5	3	2; 4	3.0	**<0.001**
	SI	79	1	3	3; 4	3.1	67	6	3	3; 4	3.0	0.559
The results of examinations and treatments.	PR	70	5	4	3; 4	3.4	68	1	3	2; 4	2.9	**<0.001**
	SI	71	4	3	3; 4	3.3	65	3	3	3; 4	3.1	0.166
Self-care: “how I should take care of myself”.	PR	69	4	4	3; 4	3.4	61	7	3	2; 4	2.7	**<0.001**
	SI	68	5	3.5	3; 4	3.3	59	8	3	3; 4	3.1	0.159
*Participation in decision making*												
I had opportunity to participate in decisions.	PR	74	4	4	3; 4	3.6	68	8	3.5	3; 4	3.2	**0.010**
	SI	74	4	4	3; 4	3.5	67	8	4	3; 4	3.3	0.227
*Caregiver’s understanding, respect, and commitment*												
Seemed to understand how I experienced my situation.	PR	77	0	4	4; 4	3.8	73	1	3	2.5; 4	3.1	**<0.001**
	SI	77	0	4	3; 4	3.6	71	2	4	3; 4	3.4	**0.046**
Was respectful towards me.	PR	74	0	4	4; 4	3.9	72	1	4	3; 4	3.4	**<0.001**
	SI	74	0	4	3; 4	3.7	70	2	4	3; 4	3.5	0.090
Showed commitment: cared about me.	PR	76	0	4	4; 4	3.9	69	1	3	2; 4	3.0	**<0.001**
	SI	76	0	4	3.25; 4	3.7	67	2	4	3; 4	3.5	**0.039**

Results from the questionnaire Quality from the Patients’ Perspective (QPP). Item scores for PR (Perceived Reality) ranged from 1 (do not agree at all) to 4 (completely agree) and for SI (Subjective Importance) from 1 (little or no importance) to 4 (of the very highest importance). NA: Not applicable. Q1; Q3: First quartile; third quartile. Statistically significant differences between groups (two-tailed *p*-value) are presented in bold font

### Outcome-related aspects

Participants in the physiotherapist-led triage group reported to a significantly higher extent that their expectations of the treatment were met, as well as the intent to follow the advice and instructions received, when compared with the standard practice group (Table 3).

**Table 3:** Outcome-related aspects of quality of care; meeting of expectations and intentions to follow advice and instructions

	Physiotherapist-led triage *n* = 83						Standard practice *n* = 80				
	*n*	NA	Median	Q1;Q3	Mean	*n*	NA	Median	Q1;Q3	Mean	*p*-value
Meeting of expectations	78	0	4	4; 5	4.3	74	0	4	3; 4	3.7	**<0.001**
Intention to follow advice and instructions	76	6	3	3; 3	2.8	59	19	3	2; 3	2.6	**0.019**

Results from the questionnaire Quality from the Patients’ Perspective (QPP). Response options ranged from 1 (not at all) to 5 (to a very large extent) for the item regarding expectations and from 1 (no) to 3 (yes, completely) for the item regarding intentions. Q1; Q3: First quartile; third quartile, NA: Not applicable. Statistically significant differences between groups (two-tailed p-value) are presented in bold font

### Missing data analysis

The missing data analyses showed significant demographic differences between those who responded to the QPP and those who did not. There was a significant difference within both groups: those who did not respond were born outside of Sweden to a higher extent (triage *p* = 0.004, standard practice *p* = 0.02). Furthermore, there were significant differences in the standard practice group: those who did not respond were younger (*p* = 0.001), lived alone (*p* = 0.007), and had a lower education level (*p* = 0.04) than those who responded.

## Discussion

This paper reports on patients’ perceptions of quality of care in a physiotherapist-led orthopaedic triage compared with standard practice. Patients in both groups reported that they perceived good quality of care, with the patients in the physiotherapist-led triage group reporting that they perceived significantly higher quality of care. This model of care seems to meet patients’ expectations and result in a greater intention to follow advice and instructions for self-management.

### Quality of care from the patient perspective

The findings in this paper are in line with previous studies reporting higher patient satisfaction with care and services received after a physiotherapist-led triage [4, 8]. We found that the patients in the triage group reported perceiving a higher medical-technical competence of the caregiver, similar to the findings of Razmjou et al. [8]. They also found significantly higher patient satisfaction regarding technical skills after physiotherapy triage compared with standard practice in a speciality shoulder clinic. Reeve et al [41] found in their qualitative study of patients’ perspectives of quality of a physiotherapist’s spinal screening service, that patients expect clinicians to be appropriately qualified and skilled.

It has previously been reported that patients expect and want information about the whole process of care, as well as provision of a diagnosis and instructions for self-management [41, 42]. The patients in the physiotherapist-led triage group in our study reported to a higher extent that they received adequate information about the whole care process. Razmjou et al. [8], also found significant differences in favour of the physiotherapist regarding explanation of the results and answers to questions. An important part of physiotherapy is education and information about symptoms and how they relate to potential underlying conditions. and it has been reported that patients attending a physiotherapist-led triage even felt less pain and anxiety upon receiving information about their problem [3]. Patients have previously reported that the most important expectation when consulting a clinician (physiotherapist or GP) is not to recover but to have their disorder confirmed [42, 43].

The patients in the physiotherapist-led triage group reported to a higher extent that the clinicians seemed to understand, respect, commit and care about them, also this in concordance with previously reported findings [8].

The results of this study suggest that this model of care is patient-centred. A narrative review and synthesis of the literature by Kitson et al. [25] identifies three core elements of patient-centred care: patient participation and involvement, relationship between the patient and the healthcare professional, and the context in which care is delivered. The authors conclude that all health professionals provide care based on these elements, but to a varying degree depending on the interest and priority given to these elements by the professional group. There is a possibility that the two different professions (physiotherapists and orthopaedic surgeons) have different responsibilities and therefore also different focus of care.

The opportunity to participate in the clinical decision-making was rated higher by the patients in the physiotherapist-led triage group. This is one of the core concepts of patient-centred care and several other studies have reported a desire of patients with musculoskeletal disorders to participate in the clinical decision-making process and feel involved in the process of the consultation [41, 42]. This could be achieved by good communication and information regarding the whole triage process. May et al. [44] found that failure to communicate effectively about the condition and the treatment options was the most frequent source of patient dissatisfaction amongst patients with back pain. It could be argued that since the primary outcome for an orthopaedic consultation is to decide whether the patient is suitable for surgery or not, there is not much room for participation. However, patients are generally less satisfied than surgeons with surgery outcomes, and one of the explanations for this is a difference in expectations of outcomes [45]. It has therefore been recommended that patients are extensively informed and to a higher extent participate in decision making [45].

Interestingly, the patients in the physiotherapist-led triage group reported to a higher extent that it was important to receive the best possible care, and that it was important that the caregiver was committed and understanding. It could be that expectations of physiotherapists differ from those of an orthopaedic surgeon; however, no studies addressing this issue have been found. Still, it has previously been reported that patients demand different quality of care depending on the care situation itself [46]. As previously mentioned, the focus of the different aspects of patient-centred care tends to vary with the professional group [25] and it could be that the expectations of patients also vary.

### Outcome-related aspects

The patients in the physiotherapist-led triage group reported to a higher extent that their expectations were met and that they were more inclined to follow the advice from the physiotherapist, which could be correlated to their care experience [28]. A clear association has previously been established between patient experience and self-rated and objectively measured health outcomes, such as adherence to recommended medication and treatments as well as use of healthcare resources [47]. Also, greater patient treatment satisfaction has been found to be associated with better adherence as well as improved persistence to recommendations or treatments [47, 48].

The finding in this study that patients in the physiotherapist-led triage group reported to a higher extent that they received useful advice on treatment and self-care, is consistent with previous research which has shown that information, exercise and pain relief are part of the physiotherapist assessment to a larger extent than assessments by other medical staff [4, 5, 49, 50]. It has been found that patients expect and wish for training programmes and advice about self-management, when seeking health care for back and neck pain [42, 43]. Considering that a large number of patients referred for orthopaedic consultation are managed non-surgically, this could be important for the patients’ wellbeing as well as further care-seeking.

### Methodological considerations

Not many studies of quality of care have used a randomised controlled design. Together with the large sample size, sufficient power and the study’s originality, this is a major strength. Furthermore, a validated satisfaction questionnaire which addresses patients’ perceptions of different aspects of care was used. The total response rate for the QPP (80 %) can be considered high, as a review of patient satisfaction with health care reported a mean response rate of 67 % for postal questionnaires [51].

Nevertheless, this study also has several limitations. There is a potential risk of performance bias in the analysis and interpretation of data since the first author was the physiotherapist performing the triage in this study, as well as responsible for the main part of the data analysis and writing of this paper. However, she was not involved in the eligibility assessment, randomisation or data collection, and all the data were coded during analysis. It could seem that the physiotherapist had a strong stake in the outcome of the study and therefore performed beyond usual care; however, the orthopaedic surgeon and all other healthcare personnel involved were briefed on the study protocol, and therefore one can argue that everyone had similar stakes and performed at their best.

The choice to use block randomisation was made to enable a balanced distribution of patients between the groups. Each block consisted of 20 envelopes and although it is a large block size which makes it harder to detect allocation sequence, there is a risk of selection bias.

Each patient consulted one of the clinicians only, which limits the possibility of investigating inter-rater agreement. Preferably, the protocol should consist of several physiotherapists and orthopaedic surgeons and also, by both healthcare professionals assessing the same patients. Due to the clinical reality at the present healthcare centre, with only one physiotherapist with appropriate level of knowledge as well as only one orthopaedic surgeon, such a protocol was not feasible. Also, on national level, many primary healthcare centres are small, and very few have orthopaedic surgeons or physiotherapists with advanced knowledge. There is a possibility that the interpersonal attributes of the therapist have influenced the outcome more than the professional roles [52]. Studies have shown that patient satisfaction with care is more related to interactions with the therapist and attributes of the therapist, than to treatment outcome [52, 53]. Due to the nature of the intervention neither the care providers nor the participants could be blinded, which might have affected the outcome.

The duration of the assessment was set according to standard practice at many clinics at the time of the study, and therefore differed between the groups (15 versus 60 min). This was done to mirror clinical practice and avoid disruptions, and to facilitate future implementation. Considering that previous research has found that having adequate time can be a determinant of satisfaction [52], the longer duration of the physiotherapist-led triage could have affected the outcome [4, 8].

Considering the minor change to the QPP questionnaire to include the physiotherapist or orthopaedic surgeon this is not likely to have affected reliability and validity of the questionnaire. Since the QPP was administrated by the Improve IT staff, the risk for detection bias is low. There is a potential risk of attrition bias due to significant demographic differences between those who responded to the questionnaire and those who did not, i.e. a risk for selective drop-out, which requires caution when interpreting the study results. Reasons for non-response are unknown. A possible reason could be that the interest in responding was low due to dissatisfaction [54]. The patients in the standard practice group were significantly older at baseline. Higher satisfaction scores from older patients have been reported in previous research [33, 34, 55] and therefore this factor cannot explain the differences in perceived care found in our study.

This study reports on patients’ perceptions of quality of care in the short term. Patient satisfaction may change based on end result, however, considering that re-visits to the GP or orthopaedic consultation were very low, as presented previously [20], it could be argued that the results were maintained over time.

The fact that only one physiotherapist and one orthopaedic surgeon performed the assessments, as well as other limitations of the study, limits generalisability of the findings and the results must be interpreted with caution.

### Future research

The aim for future research on physiotherapist-led orthopaedic triage should preferably focus on multicentre studies with several different physiotherapists and orthopaedic surgeons. While costs for physiotherapist are substantially lower than those for orthopaedic surgeons, cost effectiveness of this model should be further investigated. One problematic area is that the education as well as the professional role of physiotherapists differs internationally, which complicates generalisation. The most appropriate education for physiotherapists working in this role could be valuable to explore, and to consider for different healthcare settings.

## Conclusions

This paper reports that patients in both groups perceived good quality of care, with patients in the physiotherapist-led triage perceiving significantly higher quality of care. Additionally, this model of care seems to meet patients’ expectations and lead to a greater intention to follow advice. Our findings are in line with existing literature that this model of care provides an opportunity to shape patient-centred care that can improve access and offer care on the most appropriate level, with maintained good quality of care.

## Abbreviations

GP, General Practitioner; IO, Identity-orientated approach; MT, Medical-technical competence; NA, Not applicable; OMT, Orthopaedic Manipulative Therapy; PR, Perceived Reality; Q1; Q3, First quartile; third quartile; QPP, Quality from the Patient’s Perspective; SD, Standard Deviation; SI, Subjective importance.

## Footnotes

1 KUPP is the Swedish name for QPP
